# The PDGFBB-PDGFRβ Pathway and Laminins in Pericytes Are Involved in the Temporal Change of AQP4 Polarity during Temporal Lobe Epilepsy Pathogenesis

**DOI:** 10.1523/ENEURO.0196-25.2025

**Published:** 2025-10-24

**Authors:** Lin Lin, Hongxia Tang, Ke Cui, Zeyi Kang, Tengwei Pan, Changqiang Feng, Xiaohong Zhao, Jiewei Wang, Zhiyuan Chen, Zhengli Jiang, Gang Wu

**Affiliations:** ^1^Department of Pharmacy, Taizhou Hospital of Zhejiang Province Affiliated to Wenzhou Medical University, Linhai 317000, China; ^2^School of Pharmacy, Wenzhou Medical University, Wenzhou 325035, China; ^3^Taizhou Key Laboratory of Pharmaceuticals Therapy and Translation Research, Linhai 317000, China; ^4^Departments of Critical Care Medicine, Taizhou Hospital of Zhejiang Province Affiliated to Wenzhou Medical University, Linhai 317000, China; ^5^Neurology, Taizhou Hospital of Zhejiang Province Affiliated to Wenzhou Medical University, Linhai 317000, China

**Keywords:** AQP4 polarity, epileptogenesis, laminins, PDGFBB, pericyte, temporal lobe epilepsy

## Abstract

This study aims to examine the changes in AQP4 polarity and pericyte vascularity during temporal lobe epilepsy (TLE) progression, with the goal of identifying potential drug targets or strategies to delay the onset and progression of TLE. Chronic TLE was induced in male rats using pilocarpine. AQP4 polarity and pericyte vascular coverage were assessed by immunofluorescence. The effects of modulating AQP4 polarity on PTZ-induced TLE model using male mice were studied. Molecular mechanisms of AQP4 polarity were explored using transwell coculture and transcriptomics, validated at the protein level. ELISA was used to measure PDGF-BB levels in serum and cerebrospinal fluid. Following pilocarpine-induced chronic TLE model establishment, AQP4 polarity and pericyte vascular coverage rapidly increased but later declined, reaching the lowest levels in epileptic animals. Trifluoperazine prevented AQP4 redistribution, reduced seizure duration, and alleviated brain edema in PTZ-induced TLE mouse model. Transcriptomic analysis revealed that pericyte coculture did not alter the expression of dystrophin-associated protein components in astrocytes. Pericyte LAMA1 and LAMA2 levels were significantly higher than endothelial cells, and the levels of pericyte LAMA1 and LAMA2 were significantly increased after coculture with astrocytes. Expression of LAMA1 and LAMA2 around pericytes initially increased and then decreased during chronic TLE progression. PDGF-BB levels decreased over time, reaching the lowest levels during epilepsy. Disrupted AQP4 polarity is closely associated with TLE development. Pericyte vascular coverage appears to influence AQP4 polarity, and key molecules such as laminins and PDGF-BB may help maintain AQP4 polarity, potentially contributing to the attenuation of TLE progression and epileptogenesis.

## Significance Statement

Temporal lobe epilepsy (TLE) is a common and often drug-resistant neurological disorder. Understanding early molecular and cellular changes may reveal new treatment strategies. This study demonstrates that alterations in the polarity of aquaporin-4 (AQP4), a key water channel in the brain, are tightly linked to TLE epileptogenesis and occur in parallel with changes in pericyte vascular coverage during disease progression. We further identify laminins and PDGF-BB as pericyte-derived molecules that may help maintain AQP4 polarity. These findings highlight pericyte–astrocyte interactions as potential therapeutic targets for delaying or reducing epileptogenesis, offering novel insight into mechanisms that underlie TLE and new avenues for intervention.

## Introduction

Temporal lobe epilepsy (TLE) is the most common form of focal epilepsy and is characterized by recurrent seizures arising from the temporal lobe, often associated with synchronous abnormal brain discharges that result in behavioral abnormalities. Despite antiepileptic drug therapy, ∼30% of patients develop drug-resistant forms (Krishnamurthy, 2016); notably, in a cohort of TLE patients, up to 65% were identified as drug resistant (Bjørke et al., 2018). Common causes of epilepsy include stroke, traumatic brain injury, and status epilepticus followed by a latency period between the initial injury and the onset of epilepsy. The latent period encompasses epileptogenesis, a chain reaction of cellular and molecular changes triggered by the insults, which leads to the reorganization of neuronal networks, ultimately transforming the brain from a healthy state to an epileptic one (Lévesque et al., 2021). Investigating the pathological changes occurring during epileptogenesis in TLE and identifying therapeutic targets are crucial for advancing epilepsy management.

Aquaporin 4 (AQP4) has been implicated in the pathological processes of TLE, with altered polar distribution observed in human TLE tissue specimens (Eid et al., 2005; Binder et al., 2012). AQP4 colocalizes with Kir4.1, and changes in AQP4 polarity influence the distribution of Kir4.1, which in turn affects extracellular potassium concentrations and seizure susceptibility (Amiry-Moghaddam et al., 2003; Masaki et al., 2010; Liu et al., 2020). AQP4 forms orthogonal arrays of particles (OAPs) around blood vessels, a process dependent on dystrophin-associated protein (DAP) complex proteins, including α-syntrophin, dystrophin, and β-dystroglycan within astrocytes, as well as agrin, α-dystroglycan, and laminin extracellularly (Amiry-Moghaddam et al., 2004).

It has been reported that AQP4 is predominantly distributed on the pericyte side of blood vessels rather than on endothelial cells, as shown by immunoelectron microscopy (Gundersen et al., 2014; Hoddevik et al., 2017). Perivascular pericyte distribution changes during chronic epilepsy (Milesi et al., 2014; Arango-Lievano et al., 2018), with reduced vascular coverage and decreased AQP4 polarity observed in spontaneous epileptic animals with pericyte-specific Cdk5 knock-out (Lin et al., 2024). However, the relationship between AQP4 polarity and altered perivascular pericyte distribution remains unclear, as do the mechanisms through which pericytes regulate AQP4 polarity. It is reported that laminins in the hippocampus and cortex undergo changes in the early stages of epilepsy (Lucchi et al., 2015). Pericyte-derived factors such as laminins may influence AQP4 polarity by affecting the extracellular matrix of astrocytes or DAP complex proteins.

In this study, we dynamically analyzed the polar distribution of AQP4 and pericyte vascular coverage during temporal lobe epileptogenesis, as well as the factors mediating AQP4 polarity through pericytes. Our findings aim to identify potential therapeutic targets for TLE prevention and treatment by modulating AQP4 polarity and pericyte vascular distribution.

## Materials and Methods

### Ethics statement

The animal study was approved by the Committee for Animal Experiments at Taizhou Hospital of Zhejiang Province in China (No. K20190108). The study was conducted in accordance with the local legislation and institutional requirements.

### Pilocarpine-induced chronic TLE model

The pilocarpine model was employed for establishing chronic TLE and investigating the temporal changes in AQP4 polarity, pericyte vascular coverage, PDGF-BB, and laminins. The modeling method, adapted from reference (Lian et al., 2008), was slightly modified. Male Sprague Dawley rats (300 ± 20 g) were housed in an SPF animal facility at 21–23°C with *ad libitum* access to food and water. After 1 week of acclimation, the animals received an intraperitoneal injection of 130 mg/kg lithium chloride, followed by a subcutaneous injection of 1 mg/kg scopolamine 18 h later. After an additional 30 min, 30 mg/kg pilocarpine was administered intraperitoneally to induce status epilepticus (SE), which was terminated after 120 min with an intraperitoneal injection of 10 mg/kg diazepam. To support recovery, 2.5 ml of 5% glucose solution was administered intragastrically every 12 h for a total of five doses. All chemicals were purchased from Sigma Aldrich, except diazepam, which was obtained from Tianjin Pharmaceuticals Group.

Animals that experienced forelimb clonus or rearing and falling for at least 2 h were classified as having SE. The onset of SE was defined as persistent forelimb spasms lasting >5 min, with interruptions no longer than 2 min (Lian et al., 2008). The animals were weighed the following day. Those exhibiting SE and a weight loss exceeding 10% were included in the subsequent study. Twenty-one days after model establishment, continuous video monitoring for 1 week was used to classify animals as epileptic or nonepileptic and obtain the epilepsy frequency. Epilepsy was diagnosed when two unprovoked seizures occur after an at least 24 h time interval, in accordance with established diagnostic criteria. Among the animals that did not exhibit seizures during video monitoring, we further selected individuals with normal grooming and smooth coats, comparable with control animals, to minimize the inclusion of animals with potential delayed-onset, nonconvulsive, or subclinical epilepsy. These animals were used in PDGF-BB analysis. We acknowledge that this approach is empirical and indirect; however, it was intended to enhance the rigor of group assignment given the limitations of video-only seizure detection.

At each designated time point (end of SE, and 1, 7, or 30 d post-SE), a separate cohort of animals was anesthetized with an intraperitoneal injection of phenobarbital (30 mg/kg) for terminal blood and tissue collection. Each animal was used for sampling at only one time point. To ensure the onset of epilepsy, animals in the incubation period were selected based on the following criteria: a weight loss of over 20% at 24 h after SE, rough coat condition, and irritable behavior for 7 d following SE. Rats that received normal saline instead of pilocarpine were used as controls.

### PTZ seizure model and trifluoperazine treatment

The PTZ model was used to study the effects of modulating AQP4 polarity on acute seizure dynamics. The modeling method was slightly modified from a previously described protocol (Shen et al., 2016). Male C57BL/6 mice (25 ± 5 g) were selected for the study. PTZ (60 mg/kg) was administered intraperitoneally, and the trifluoperazine treatment group received an intraperitoneal injection of trifluoperazine (30 mg/kg) simultaneously with PTZ. Seizure latency and duration were observed and recorded for 30 min. Seizures were classified according to the Racine scale, with grade 2 seizures representing macroscopically visible epileptic symptoms (Racine, 1972). Three hours after PTZ administration, the animals were killed by intraperitoneal injection of a high dose of phenobarbital (over 150 mg/kg). The brains were harvested, weighed wet, and then dried at 110°C for 24 h. Brain tissue water content was calculated using the formula: Water Content (%) = [(Wet Weight − Dry Weight) / Wet Weight] × 100%. As both pilocarpine and PTZ models primarily recapitulate TLE features, our study focuses specifically on TLE-related pathological changes.

### Immunofluorescence

The immunofluorescent assay was conducted as previously described (Lin et al., 2024). Briefly, mice were anesthetized, perfused with PBS and 4% paraformaldehyde, and their brains were extracted. The brains were fixed in 4% paraformaldehyde for 6 h, transferred to 30% sucrose in PBS, and sectioned at 20 µm using a cryostat (Leica CM1950).

Before staining, sections were washed three times with PBS, permeabilized with 1% Triton X-100 for 15 min, blocked with donkey serum for 1 h, and incubated at 4°C for 2 d with the following primary antibodies: AQP4 (MilliporeSigma, A5971), PDGFRβ (Abcam, ab32570), Glut1 (Abcam, ab40084), LAMA1 (Invitrogen, MA1-21194), or LAMA2 (Santa Cruz, Sc-59894). After washing three times with PBS, fluorescent secondary antibodies were applied and incubated in the dark for 4 h. Sections were then washed again three times with PBS, stained with 5 µM DAPI solution for 15 min, washed three more times, mounted with anti-fade reagent, and imaged using confocal laser scanning microscopy (Zeiss LSM800).

For the pericyte vascular coverage and LAMA1/LAMA2 assays, Z-stack scanning was performed. In the AQP4 polarity assay, uniform high and low stringency thresholds were applied to all images. Low stringency thresholds defined the overall AQP4 immunoreactivity region, while high stringency thresholds defined AQP4 signals localized to perivascular end feet. The AQP4 polarity index was calculated as the ratio of fluorescence intensity at high versus low thresholds, with higher values indicating greater polarity distribution (Wang et al., 2012). Pericyte vascular coverage was determined by dividing the PDGFRβ-positive area by the Glut1-positive area in the perivascular zone using Z-projection analysis (Lin et al., 2024). Image analysis was performed with ImageJ software.

### PDGF-BB assay

Animals were secured in a stereotaxic apparatus. After separating the neck skin and muscles, the medullary cistern was exposed, and cerebrospinal fluid (CSF) was carefully aspirated using a 33G syringe under a stereomicroscope (RWD Life Science, model 77001) and transferred to an EP tube. Blood was collected via orbital sampling, and serum was separated by centrifugation at 1,000 × *g* for 15 min following a 3 h incubation at room temperature. PDGF-BB levels were measured using an ELISA kit (R&D Systems, MBB00) according to the manufacturer’s instructions.

### Cell culture

Transwell coculture experiments were performed with HA1800 (Shenzhen Huatuo), HBVP (Zhongqiao Xinzhou Biotechnology, ZQ0993), and hCMEC (Zhongqiao Xinzhou Biotechnology, ZQ0961). Six-well transwell plates (Falcon, PET membrane 1.0 μm pore size, catalog #FAL-353502/353102) were used with 5 × 10^5^ cells in the upper and lower layers and 10% FBS in DMEM medium. The Trasnwell inserts were inverted, and the lower cells were cultured on the membrane, placed upright into the culture plate 24 h later, and the upper cells were added for culture, with a transwell insert medium volume of 3 ml. After 48 h of transwell culture, cells on both sides of the membrane were gently scraped with a cell scraper to extract total protein, membrane protein (Thermo Fisher Scientific, Mem-PER Plus Membrane Protein Extraction Kit), or RNA from upper and lower cells for experiments.

### Transcriptomics experiment

Both the up and down cells of HA1800-HA1800, HA1800-HBVP, HA1800-hCMEC, and HBVP-HBVP transwell chamber (cell before “-” is the up layer, cell after “-” is the lower layer) were collected for high-throughput mRNA sequencing. Total RNA was extracted using a TRIzol reagent (Invitrogen) followed by quality check on a NanoDrop spectrophotometer (Thermo Fisher Scientific), and a 1% agarose gel. Qualified samples, which had an RNA integrity number >7, were retained for next-generation library preparation according to the manufacturer’s protocol (an NEBNext Ultra RNA Library Prep Kit for Illumina). The resulting RNA was reverse transcribed into cDNA using a cDNA synthesis kit (Takara, RR036A). After that, DNA sequencing of the libraries was performed on an Illumina sequencer, and data were analyzed by GENEWIZ.

### Western blot (WB)

The brain hippocampus and cells were extracted and then homogenized or lysized in lysis buffer containing a protease inhibitor cocktail. The supernatant was collected after centrifugation at 12,000 rpm for 10 min. The cells in the up or down layer of transwell chamber were collected and the total or membrane protein were extracted according to the manufactory protocol. The protein concentration was determined using the rapid gold BCA protein assay kit (Thermo Fisher Scientiﬁc). Equivalent amount of protein was subjected to SDS-PAGE gel (6–10%). The entire blot was cropped according to the molecular weight specified in the antibody datasheets. The cropped blots were separately transferred to PVDF membranes (Millipore) and then probed with anti-AQP4 (CST, 59678), anti-LAMA1 (Novus, NB300-144), LAMA2 antibody (Santa Cruz, Sc-59854), anti-GAPDH (abcam, ab181602), β-actin (Abway, AB0035), or anti-Vinculin (Proteintech, 66305-1-Ig) at 4°C overnight and then incubated with HRP conjugated secondary antibodies. The proteins were visualized by an enhanced chemiluminescence detection system. For quantification, only the bands at the molecular weight indicated in the antibody datasheet for each target protein were selected. The density of protein bands was quantified using ImageJ software (US National Institutes of Health) and normalized to GAPDH, β-actin, or vinculin from the same blot.

### Statistical analysis

The data are presented as mean ± SEM. Statistical analyses were performed using GraphPad Prism 9 (GraphPad Software). Group differences were evaluated using one-way ANOVA, followed by Sidak’s post hoc test for multiple comparisons. A significance level of *p* < 0.05 was considered statistically significant.

## Results

### Changes in AQP4 polarity during the pathological process of TLE

**Figure 1.**
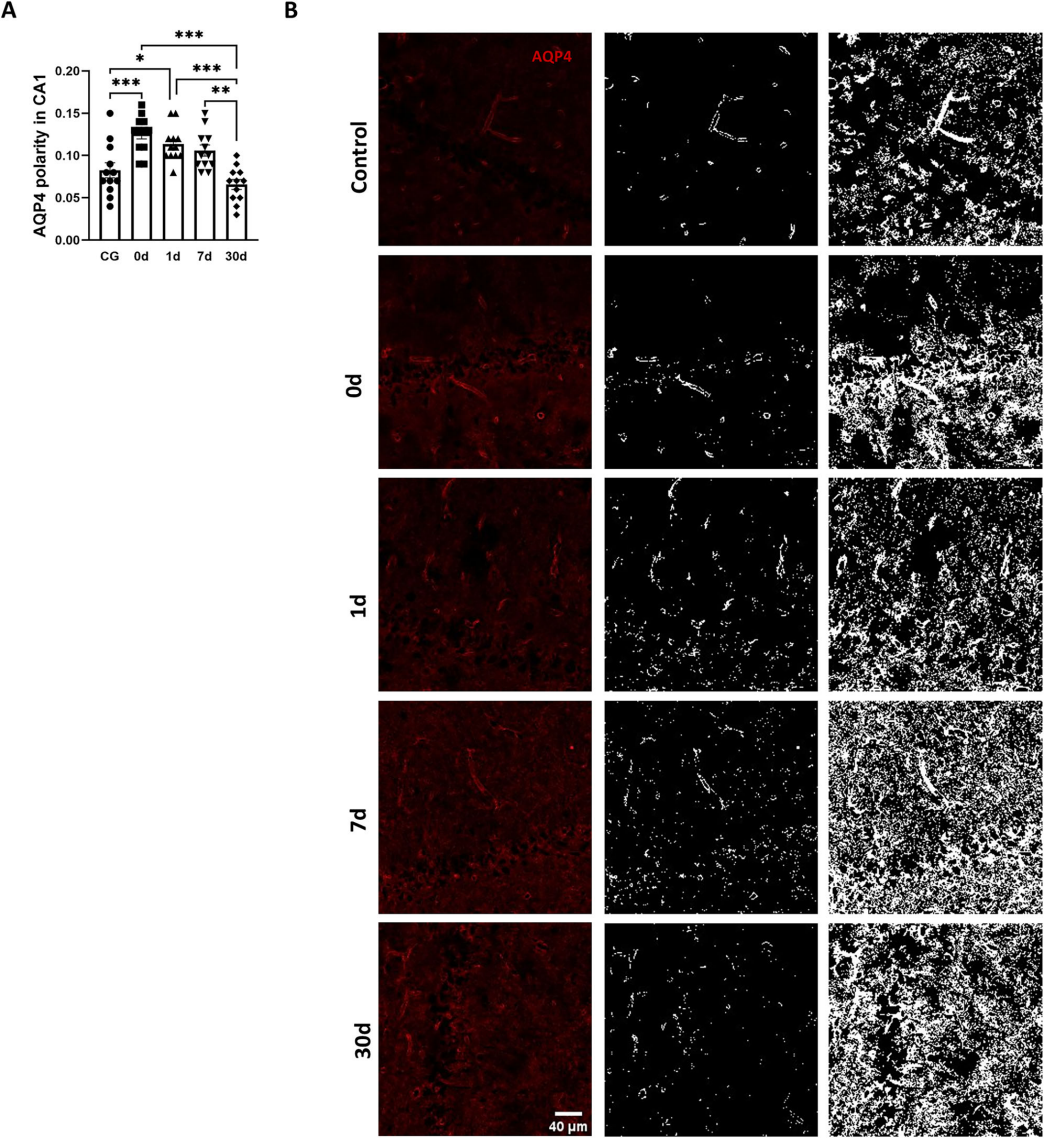
Temporal change of AQP4 polarity during the process of pilocarpine-induced TLE. ***A***, Quantitative analysis of AQP4 polarity in control group (CG), 0 d, 1 d, 7 d, 30 d after SE. *n* = 12, 3 rats per group, 4 slice per rat, ***p < 0.001. ***B***, Representative immunofluorescence images (red, AQP4; blue, DAPI) and gray images of AQP4 at high and low stringency to calculate the AQP4 polarity. Scale bar, 40 μm. 0 d represents the time point immediately after the termination of pilocarpine-induced SE.

We used pilocarpine to create a rat model of chronic TLE and observed changes in AQP4 polarity during the pathological process. We used 50 rats to establish a model of sustained epilepsy. Three rats did not develop SE, three died shortly after the onset of SE, four died 1 d after SE, and two died within 1–7 d post-SE. Ultimately, the groups were divided as follows: eight animals in the immediate post-SE termination (0 d), 1 d, 7 d, and 30 d nonepilepsy groups and six animals in the 30 d epilepsy group. The control group consisted of eight animals. The epilepsy frequency for the 30 d epilepsy group was 3.83 ± 0.76 seizures per week. Immunofluorescence results showed that AQP4 polarity in the CA1 region of the hippocampus was significantly increased at 0 and 1 d after SE compared with the control group, and the mean value at 7 d after SE was higher than that of the normal control group, but there was no significant difference (Fig. 1*A*,*B*). AQP4 polarity in epileptic animals at 30 d after model establishment was significantly lower than that at 0, 1, and 7 d, and the mean value was slightly lower than that in the control group, but there was no significant difference (Fig. 1*A*,*B*).

### Inhibition of AQP4 polar distribution mitigates PTZ-induced seizures

**Figure 2.**
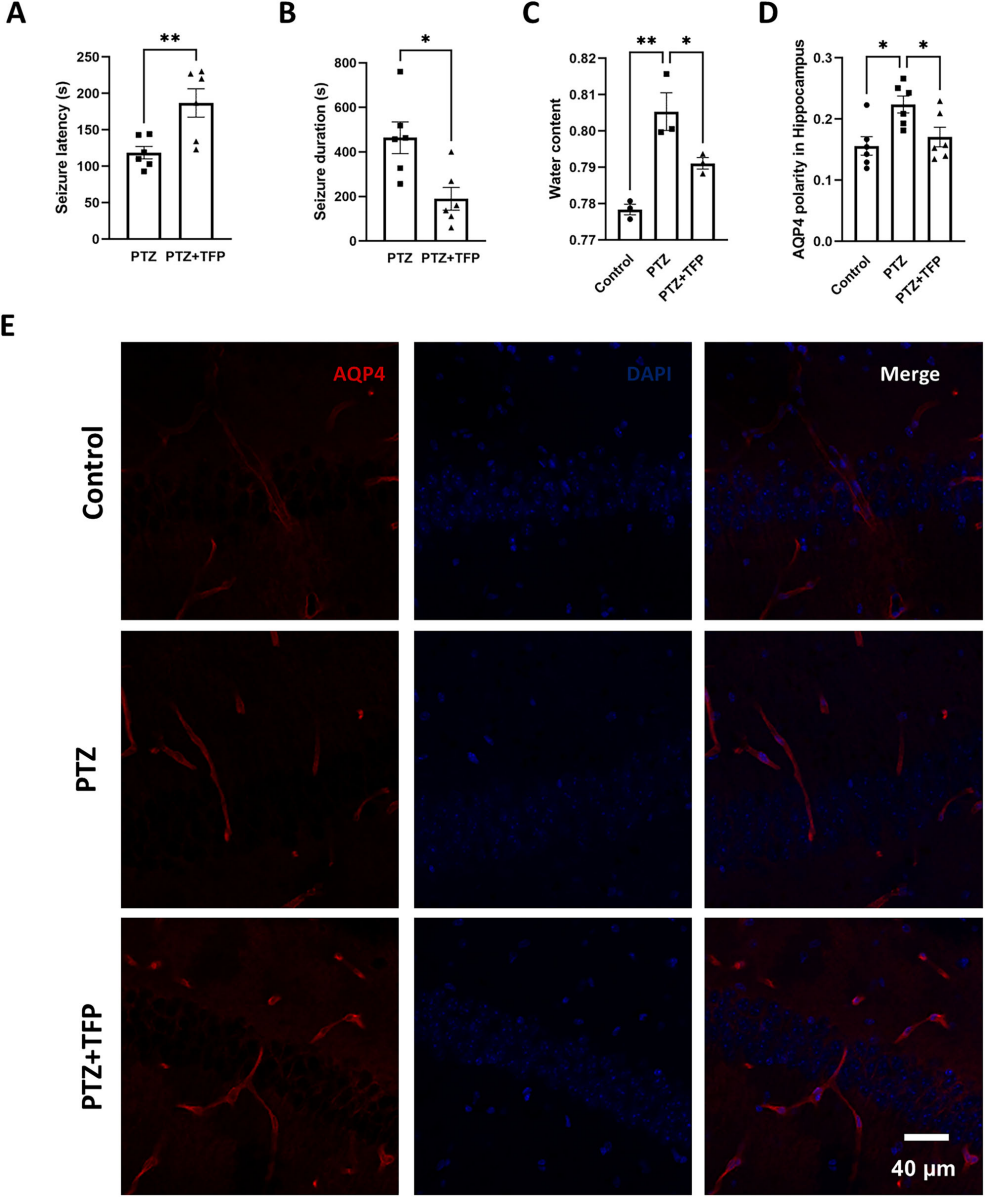
Effect of the AQP4 polarity distribution inhibitor trifluoperazine on PTZ-induced seizures: ***A***, Seizure latency. ***B***, Seizure duration (*p < 0.05, *n* = 6 per group). ***C***, Brain edema 3 h after PTZ injection (*p < 0.05, **p < 0.01, *n* = 3 per group). ***D***, Statistical analysis of hippocampal AQP4 polarity distribution across groups (**p < 0.01, *n* = 3 animals per group, 2 slices per animal). ***E***, Representative immunofluorescence images: red, AQP4; green, Glut1; blue, DAPI. Scale bar, 40 μm. Trifluoperazine, TFP.

To investigate the relationship between AQP4 polarity distribution and TLE, we investigated the effect of trifluoperazine, a calmodulin antagonist which was reported to be able to inhibit polar distribution of AQP4 (Kitchen et al., 2020), on PTZ-induced seizures. The results showed that trifluoperazine treatment coinciding with PTZ administration was able to prolong PTZ-induced seizure latency (Fig. 2*A*), inhibit seizure duration (Fig. 2*B*), and simultaneously inhibit brain edema resulting from seizures (Fig. 2*C*). PTZ-induced seizures were accompanied by an increase in AQP4 polarization, and this effect appeared to be attenuated by trifluoperazine (Fig. 2*D*,*E*). These findings suggested a possible association between AQP4 polarity and seizure severity.

### Changes in pericyte vascular coverage during TLE epileptogenesis

**Figure 3.**
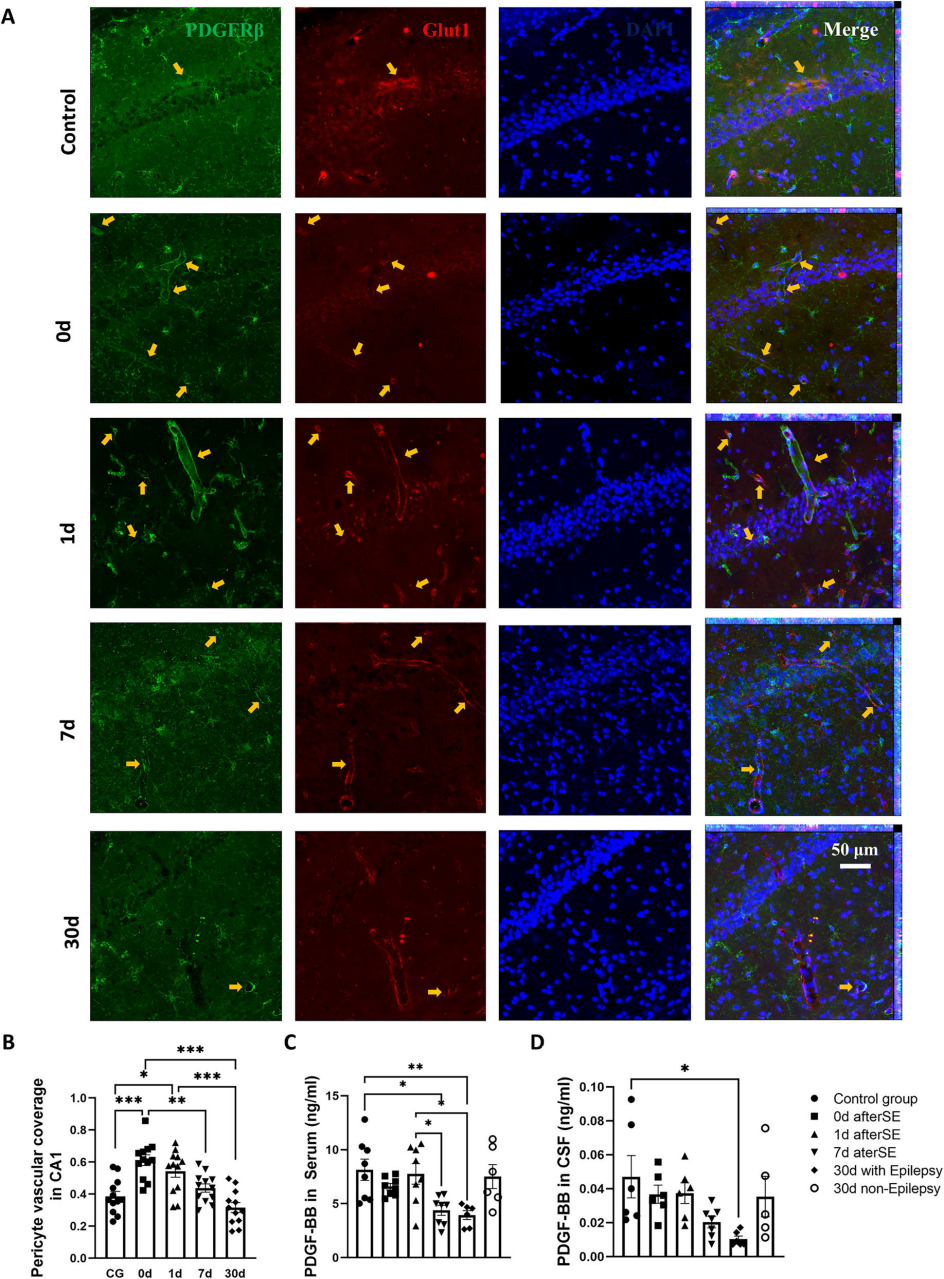
Changes in pericyte vascular coverage and PDGF-BB concentration in chronic TLE rats induced by pilocarpine. ***A***, Representative immunofluorescence images of pericyte vascular coverage: red, Pdgfrβ; green, Glut1; blue, DAPI. Scale bar, 50 μm. Yellow arrows indicate pericytes surrounding blood vessels. ***B***, Statistical analysis of pericyte vascular coverage in the hippocampal CA1 region. *n* = 12, 3 animals per group, 2 brain slices per animal, 2 hippocampal regions. *p < 0.05, **p < 0.01, ***p < 0.001. ***C***, PDGF-BB concentration in serum. *n* = 8 for control group (CG), 0 d, 1 d, 7 d; *n* = 6 for 30 d with or without epilepsy. *p < 0.05, **p < 0.01. ***D***, PDGF-BB concentration in CSF. *n* = 6 for control group, 0 d, 1 d, 30 d with epilepsy; *n* = 7 for 7 d; *n* = 5 for 30 d nonepilepsy. *p < 0.05.

The temporal change of pericyte vascular coverage was studied using the pilocarpine-induced chronic TLE rat model. The results of Z-stack immunofluorescence with PDGFRβ and Glut1 costaining showed that the vascular coverage rate of pericytes in the hippocampus increased after modeling, but gradually decreased thereafter. The vascular coverage rate of pericytes at 0 and 1 d after SE was significantly higher than that of the normal control group, and at 7 d after SE was significantly lower than that at 0 d after SE. Pericyte vascular coverage in epileptic animals at 30 d after SE was significantly lower than that at 0 and 1 d after SE, and the mean value was lower than that in normal controls and 7 d after SE, but there was no significant difference (Fig. 3*A*,*B*). Compared with the results of the second part, pericyte vascular coverage was consistent with changes in AQP4 polarity during TLE epileptogenesis.

### Changes of PDGF-BB during the pathological process of TLE

It has been reported that PDGF-BB can promote the distribution of pericytes around blood vessels, and we used ELISA to detect the content of PDGF-BB in serum and CSF of pilocarpine-induced chronic TLE rats. Of the eight animals that showed no seizures during video monitoring, six with normal grooming and smooth coats—similar to control animals—were selected for the PDGF-BB assay. The content of PDGF-BB in serum significantly decreased at 7 d after SE and in epileptic animals at 30 d compared with the normal group and 1 d after SE. The content of PDGF-BB in serum in 0 d, 1 d, and nonepileptic animals at 30 d showed no difference to normal control (Fig. 3*C*). The content of PDGF-BB in CSF decreased gradually after model establishment, and only epileptic animals at 30 d after SE were lower than normal controls (Fig. 3*D*). Decreased vascularity of pericytes in pilocarpine-induced TLE animals was associated with gradually decreased levels of PDGF-BB.

### Pericyte culture enhanced the membrane distribution of AQP4

**Figure 4.**
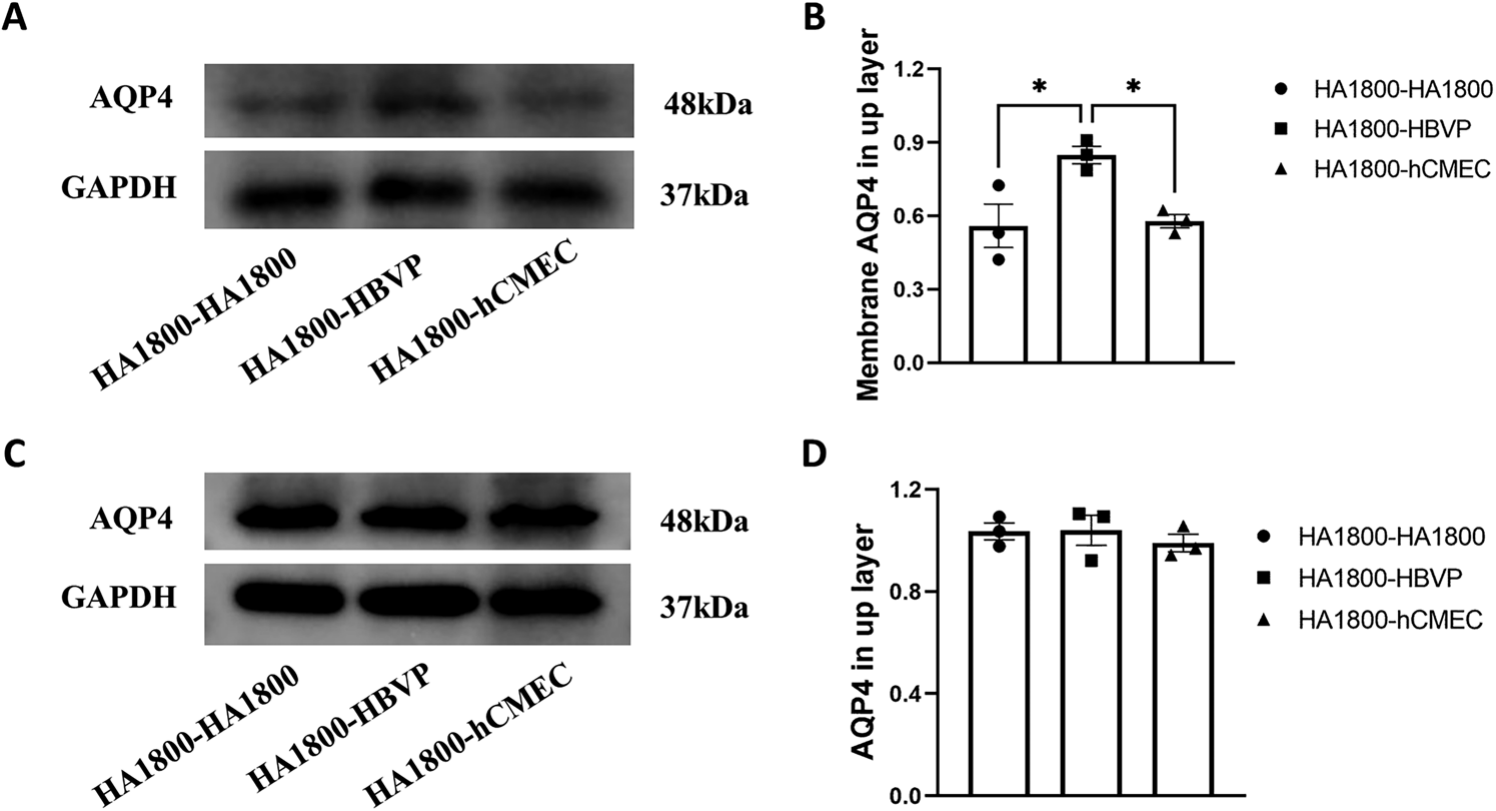
Effect of pericyte coculture on astrocyte membrane AQP4 levels. HA1800, HBVP, and hCMEC were transwell cocultured with HA1800 cells. AQP4 levels in the membrane of upper layer of HA1800 cell: ***A***, Representative WB images. ***B***, Statistical analysis, *n* = 3, *p < 0.05. The total AQP4 levels in the upper layer of HA1800 cells: ***C***, representative WB images, ***D***, statistical analysis, *n* = 3. The uncropped Western blots see Extended Data Figure 4-1.

To investigate whether the distribution of AQP4 was associated with pericyte distribution, we cocultured astrocyte HA1800, pericyte HBVP, and endothelial cell hCMEC with HA1800 in transwells to detect the expression of AQP4 total protein and AQP4 in the cell membrane of upper HA1800 cells. WB results showed that HBVP could promote the increase of AQP4 content on the cell membrane compared with HA1800 and hCMEC (Fig. 4*A*,*B*) but had no effect on the total amount of AQP4 (Fig. 4*C*,*D*; Extended Fig. 4-1). Thus, pericyte distribution promotes membrane distribution of AQP4.

10.1523/ENEURO.0196-25.2025.f4-1 Figure 4-1 Original Western blots in Figure 4. The quantified bands correspond to the molecular weight indicated in the antibody datasheet of AQP4 and GAPDH. Download Figure 4-1, TIF file.

### Pericyte laminins may mediate AQP4 polarity

**Figure 5.**
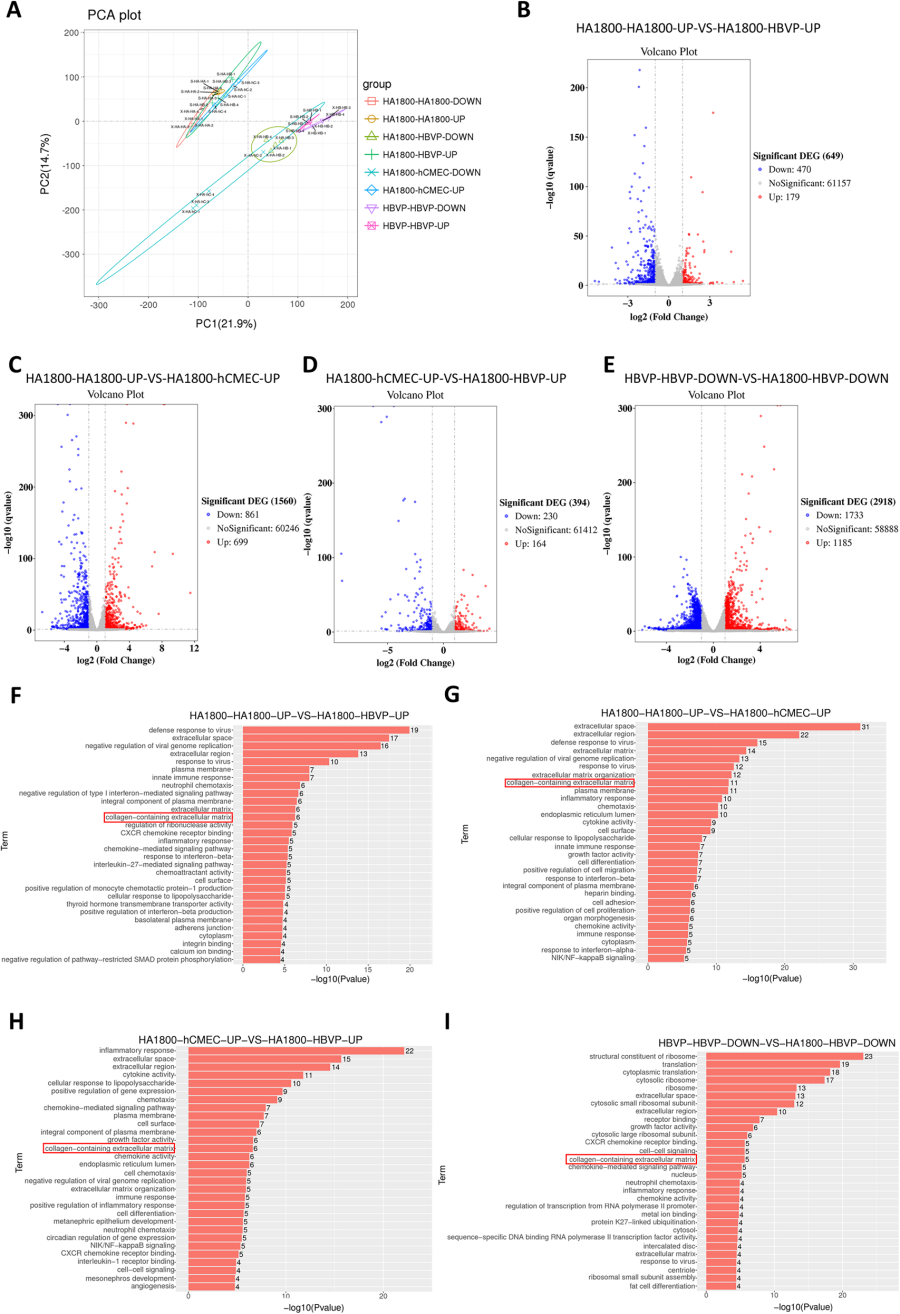
Transcriptomic analysis of lower and upper layer cells after transwell coculture of HA1800, HBVP, hCMEC with HA1800, and HBVP with HBVP cells. Each group contains four specimens. ***A***, Principal component analysis plot. ***B***, Volcano plot of differentially expressed genes (DEG) comparing upper layer HA1800 in HA1800-HBVP to HA1800-HA1800 coculture. ***C***, Volcano plot of DEG comparing upper layer HA1800 in HA1800-hCMEC to HA1800-HA1800 coculture. ***D***, Volcano plot of DEG comparing upper layer HA1800 in HA1800-HBVP to HA1800-hCMEC coculture. ***E***, Volcano plot of DEG comparing lower layer HBVP in HA1800-HBVP to HBVP-HBVP coculture. ***F–I***, GO analysis of DEG in ***B–E***, respectively. The analysis results of AQP4 polarity-related genes associated with GO term “collagen-containing extracellular matrix” is presented in Extended Data Figure 5-1.

To search for factors mediating AQP4 distribution in pericytes, we performed transwell experiment with HA1800, HBVP, hCMEC and HA1800, HBVP and HBVP and collected cells for transcriptomic sequencing analysis after cell culture (Fig. 5). Transcriptome results showed that pericyte coculture resulted in decreased transcription of 470 genes and increased transcription of 179 genes in astrocytes (Fig. 5*B*). Endothelial cell coculture resulted in decreased expression of 861 genes and increased transcription of 699 genes in astrocytes (Fig. 5*C*). Endothelial cells coculture resulted in downregulation of 230 genes and upregulation of 164 genes in astrocytes compared with pericytes coculture (Fig. 5*D*). In parallel, astrocyte cultures increased pericyte transcription of 1,185 genes and decreased transcription of 1,733 genes (Fig. 5*E*). By GO analysis, collagen-containing extracellular matrix was found to be involved in the transcriptional changes of genes in pericytes, endothelial cells, and astrocytes coculture (Fig. 5*F–I*), so we performed a specific analysis of this GO genes.

10.1523/ENEURO.0196-25.2025.f5-1 Figure 5-1 Results of differential expression analysis of AQP4 polarity-related genes associated with the GO term “collagen-containing extracellular matrix”. Download Figure 5-1, XLS file.

**Figure 6.**
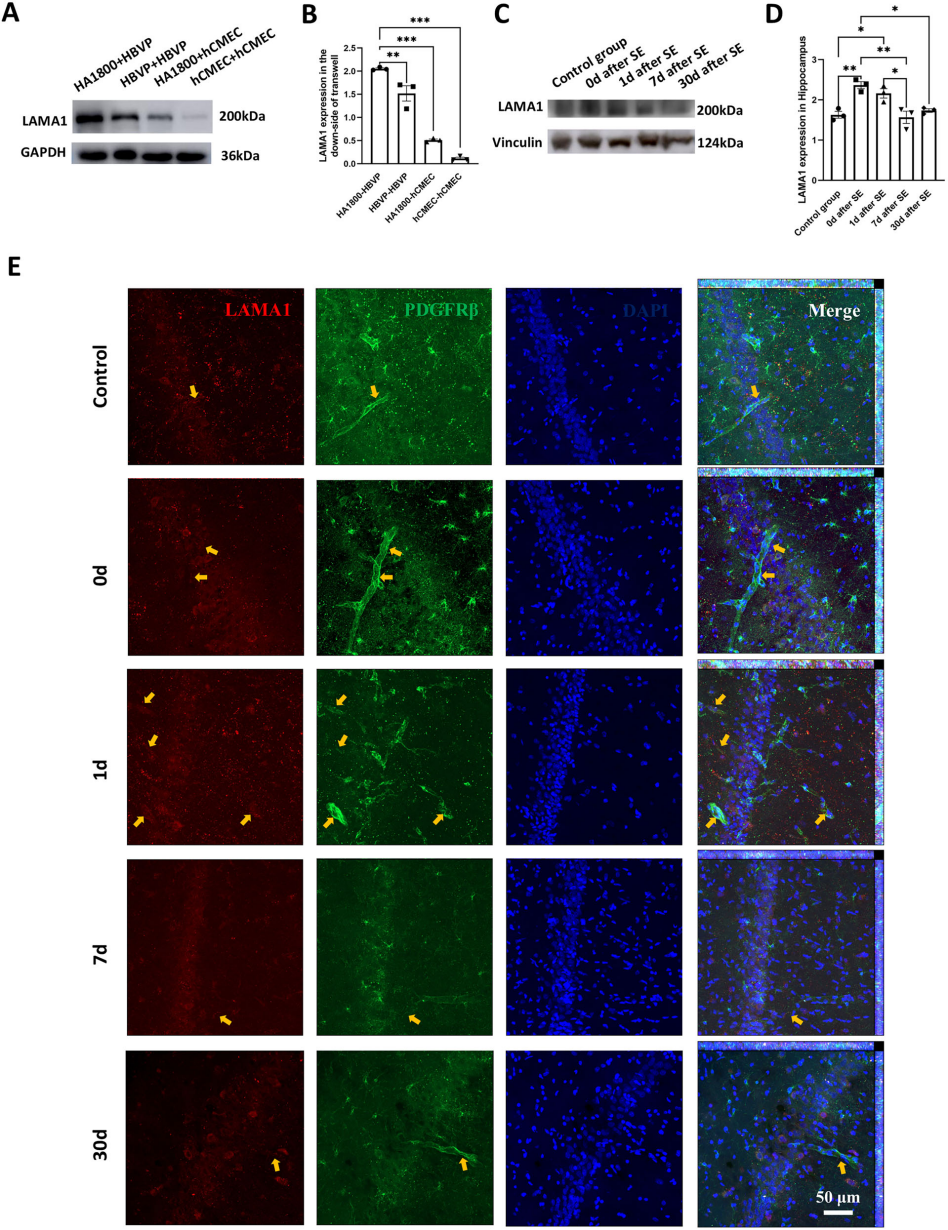
WB analysis of LAMA1 in lower layer HBVP and hCMEC cells after transwell coculture of HA1800-HBVP, HBVP-HBVP, HA1800-hCMEC, and hCMEC-hCMEC: Representative WB image (***A***) and statistical analysis (***B***) of LAMA1 expression, *n* = 3, **p < 0.01, ***p < 0.001. Changes in hippocampal LAMA1 in chronic TLE rats induced by pilocarpine: representative WB image (***C***) and statistical analysis (***D***), *n* = 3 animals per group, *p < 0.05, **p < 0.01. The uncropped Western blots, see Extended Data Figure 6-1. ***E***, Representative immunofluorescence images of LAMA1 in each group. Red, LAMA1; green, PDGFRβ; blue, DAPI. Yellow arrows indicate LAMA1 localized surrounding pericytes. Scale bar, 50 μm.

**Figure 7.**
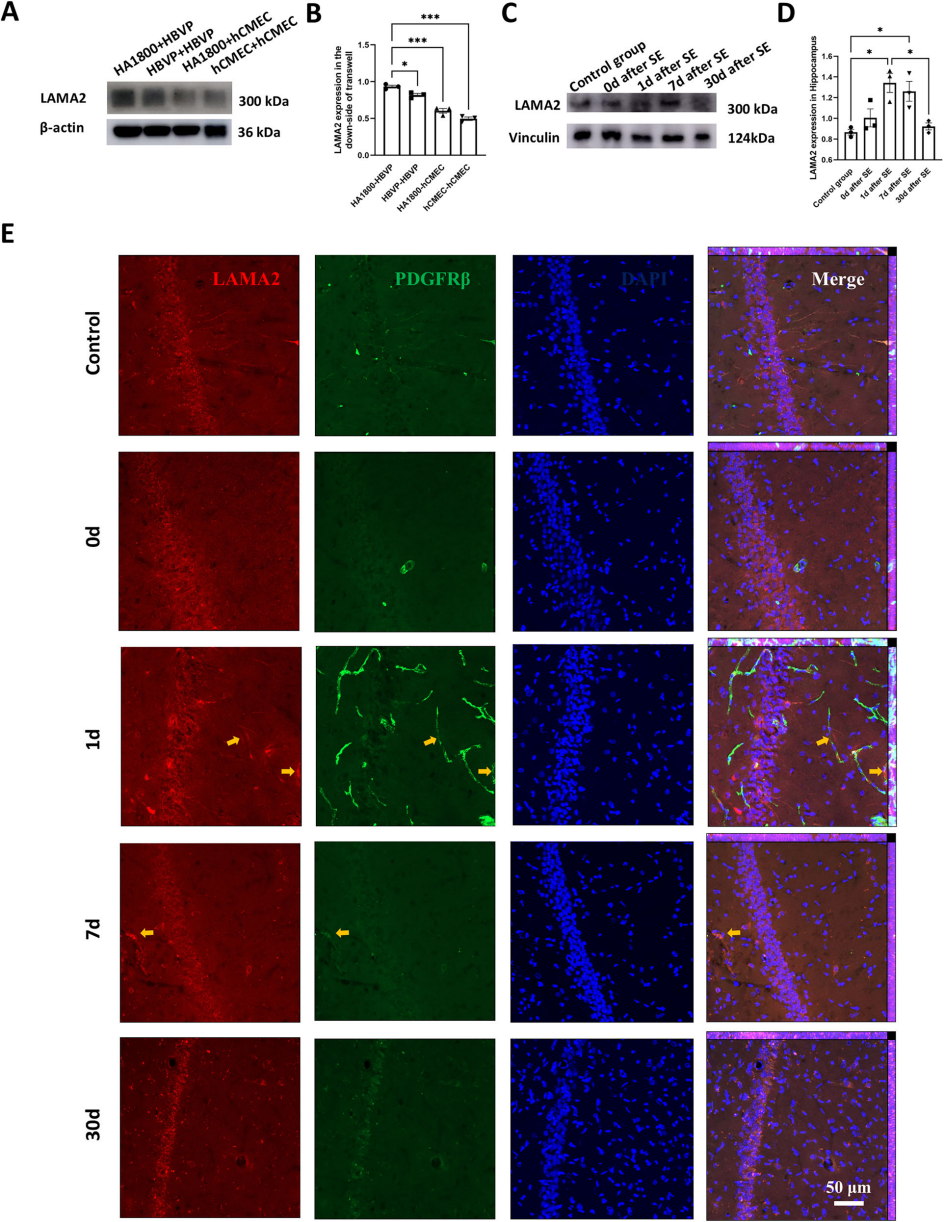
Analysis of LAMA2 expression in transwell cocultures and hippocampal tissue from pilocarpine-induced chronic TLE rats: ***A***, representative WB image and ***B***, statistical analysis of LAMA2 expression in lower-layer HBVP and hCMEC following transwell coculture of HA1800-HBVP, HBVP-HBVP, HA1800-hCMEC, and hCMEC-hCMEC (*n* = 3, *p < 0.05, ***p < 0.001). ***C***, Representative WB image and ***D***, statistical analysis of hippocampal LAMA2 expression in chronic epilepsy rats induced by pilocarpine (*n* = 3 animals per group, *p < 0.05). The uncropped Western blots, see Extended Data Figure 7-1. ***E***, Representative immunofluorescence images of LAMA2 expression in each group. Red, LAMA2; green, PDGFRβ; blue, DAPI. Yellow arrows highlight LAMA2 localization around pericytes.

The result showed that (1) AQP4 and its polarity-associated proteins, such as α-syntrophin (SNTA1), agrin (AGRN), dystrophin (DMD, dystrophin-protein71, DP-71), β-dystroglycan (DAG1), and α-dystrobrevin (DTNA) in astrocyte were not changed after cocultured with pericyte compared with endothelial cells, while IL-33 (cytosolic DNA-sensing pathway), ICAM1, CDH5, ITGB3, and LAMC2 were decreased and PDGFB was increased. (2) Astrocyte coculture induced increased LAMA1, LAMA2, PDGFRB, PDGFB, and LAMC1, and decreased LAMB3, LAMC2, LAMA3, LAMC3, and AGRN in pericyte compared with endothelial cells. (3) Pericyte transcripted higher PDGFRB, PDGFB, LAMA2, LAMA1, LAMC1, LAMB1, LAMC2, and LAMA3 and lower LAMA5, LAMB3, and AGRN compared with astrocyte after astrocyte coculture (Extended Data Fig. 5-1). Transcriptome results indicated that pericytes’ laminins might be involved in astrocyte AQP4 distribution, and we performed WB validation on cocultured cells. The results showed that pericyte LAMA1 levels were significantly higher than endothelial cells, and the levels of pericyte LAMA1 were significantly increased after coculture with astrocytes (Fig. 6*A*,*B*; Extended Data Fig. 6-1). LAMA2 also showed the same changes (Fig. 7*A*,*B*; Extended Data Fig. 7-1), which is consistent with the results of transcriptomics.

10.1523/ENEURO.0196-25.2025.f6-1 Figure 6-1 Original Western blots in Figure 6. The quantified bands correspond to the molecular weight indicated in the antibody datasheet of LAMA1. Download Figure 6-1, TIF file.

10.1523/ENEURO.0196-25.2025.f7-1 Figure 7-1 Original Western blots in Figure 7. The quantified bands correspond to the molecular weight indicated in the antibody datasheet of LAMA2. Download Figure 7-1, TIF file.

### Changes of laminins during TLE epileptogenesis

To investigate whether laminins are associated with altered AQP4 polarity during TLE progression, we performed temporal immunofluorescence detection of laminins in hippocampal tissues of pilocarpine-induced chronic TLE rats. The results revealed that hippocampal LAMA1 levels increased significantly immediately after SE and then gradually decreased. One day after model establishment, LAMA1 levels remained elevated compared with the control group but decreased significantly by Day 7, returning to control levels. At 30 d, LAMA1 levels were significantly lower than those observed immediately after SE and on Day 1 (Fig. 6*C–E*; Extended Data Fig. 6-1). In contrast, LAMA2 levels did not increase immediately after epilepsy but showed significant elevation at Days 1 and 7, returning to control levels by Day 30 (Fig. 7*C–E*; Extended Data Fig. 7-1). These trends paralleled changes in AQP4 polarity and pericyte vascular coverage. While LAMA1 and LAMA2 are not exclusively expressed in pericytes, their expression around pericytes increased proportionally with their overall expression levels (Figs. 6*E*, 7*E*).

## Discussion

This study investigated whether AQP4 polarity during the TLE pathological process is influenced by the vascular distribution of pericytes, focusing on temporal changes. The findings revealed that alterations in AQP4 polarity closely align with the vascular coverage of pericytes in epilepsy. Furthermore, LAMA1 and LAMA2 laminins in pericytes were shown to influence AQP4 distribution in astrocytes. Changes in AQP4 polarity during epilepsy were also correlated with laminin expression. To our knowledge, this is the first study to identify a link between astrocyte AQP4 polarity and pericyte vascularization during TLE, highlighting the potential regulatory role of pericyte vascular coverage and laminins in this process.

Nearly 30% of epilepsy patients are unresponsive to medications (Krishnamurthy, 2016), highlighting the importance of early intervention in high-risk individuals before symptom onset. Understanding the pathophysiologic changes during epileptogenesis is crucial to identifying potential therapeutic targets, drugs, or modulators that can inhibit this process (Shariff et al., 2024). While neuronal damage is a key focus, the pathological roles of non-neuronal elements, including astrocytes, vascular neural units, and other cell types, also warrant significant attention (Janigro and Walker, 2014).

The astrocytic water channel protein AQP4 plays a critical role in epileptic pathogenesis including TLE (Binder et al., 2012). Modulating AQP4 may serve as an effective therapeutic target for TLE. AQP4 colocalizes with Kir4.1 (Masaki et al., 2010), and alterations in its polarized distribution influence potassium (K^+^) ion homeostasis, a key factor in epileptogenesis. In the hippocampus, astrocytic AQP4 and Kir4.1 cooperate in K^+^ regulation, and depletion of the perivascular AQP4 pool slows K^+^ clearance, exacerbating seizure severity (Amiry-Moghaddam et al., 2003). Here, the PTZ-induced seizure experiment suggested a possible association between AQP4 polarity and seizure severity in TLE. In human epileptic hippocampal tissues, AQP4 polarization is significantly reduced compared with normal tissues (Eid et al., 2005; Peixoto-Santos et al., 2017). Notably, our results found that, in TLE animals, AQP4 polarization initially increased during the early stages of acute injury but decreased progressively, reaching its lowest levels in chronic epilepsy. To our knowledge, this is the first study to explore the dynamics of AQP4 polarization throughout the TLE process. These findings underscore the need to consider the stage of epilepsy when targeting AQP4 polarization for therapeutic intervention.

Based on these results, the search for regulators of AQP4 polarity distribution may offer a promising strategy to delay TLE epileptogenesis. Immunoelectron microscopy has revealed that AQP4 distribution is more pronounced around pericytes than endothelial cells, with pericytes mediating AQP4's polar distribution (Gundersen et al., 2014; Hoddevik et al., 2017). Consistently, the present study found that coculture with pericytes significantly increased AQP4 expression on astrocyte membranes. In Pdgfb^ret/ret^ transgenic mice (Armulik et al., 2010), pericyte LAMC1-knock-out animals (Gautam et al., 2016), and pericyte Cdk5 knock-out spontaneously epileptic mice (Lin et al., 2024), both AQP4 polarity distribution and pericyte vascular coverage were concurrently reduced, underscoring a potential correlation. However, the temporal dynamics of these changes during the pathological progression of epilepsy remain unexplored.

In this study, pericyte vascular coverage increased in the early stages after SE injury and progressively decreased as TLE advanced, with the lowest expression observed in epileptic animals. Consistent with our findings, previous research reported increased pericyte vascular coverage in PDGFRβ^+^ cells during the acute phase after SE (Milesi et al., 2014). However, decreased vascular coverage was noted in NG2-positive cells (Milesi et al., 2014; Arango-Lievano et al., 2018). This discrepancy may be due to the distinct roles of NG2 and PDGFRβ in characterizing vascular mural cells, as NG2 primarily marks oligodendrocyte precursor cells (Bottero et al., 2024), whereas PDGFRβ preferentially identifies pericyte cells (Naranjo et al., 2022), despite also being expressed in nonvascular brain parenchymal cells. In human brain tissue with chronic TLE, one study reported individual variability in PDGFRβ vascular distribution (Milesi et al., 2014). Another study observed an increased number of blood vessels fully encapsulated by PDGFRβ-positive cells, alongside a decrease in incompletely encapsulated vessels, indicating increased pericyte coverage in epileptic tissues (Liu et al., 2024). However, this study focused on the cerebral white matter of the temporal gyrus, differing from our investigation of the hippocampal region, which may explain the discrepancies. In summary, our study unraveled the dynamic changed of vascular coverage of PDGFRβ-positive pericytes in the hippocampal tissue of rats with TLE progression. Notably, the perivascular distribution of pericytes closely paralleled changes in AQP4 polarity during TLE progression. This suggests that pericytes may play a critical role in regulating AQP4 polarity distribution in astrocytes.

The polar distribution of AQP4 is influenced by DAP complex proteins, including β-DG, syntrophin, and extracellular matrix components (Amiry-Moghaddam et al., 2004; Waite et al., 2012). Using transwell transcriptomics, we observed no changes in the expression of AQP4, α-syntrophin, agrin, dystrophin, β-dystroglycan, or α-dystrobrevin in astrocytes cocultured with pericytes compared with endothelial cells. In line with single-cell sequencing data (Vanlandewijck et al., 2018), we found significantly higher expression of laminins, including LAMA1, LAMA2, and LAMC1, in pericytes than in endothelial cells. Coculture with astrocytes increased LAMA1 and LAMA2 expression in pericytes, suggesting that laminins like LAMA1 in pericytes may play a key role in AQP4 distribution. This is evidenced by the fact that the interaction between laminins and dystroglycan is critical for maintaining the OAPs and polarity of AQP4 (Amiry-Moghaddam et al., 2004; Waite et al., 2012) and knock-out of LAMC1 in pericytes reduces AQP4 polarity distribution (Gautam et al., 2016). In this study, the levels of LAMA1 and LAMA2 increase in response to seizures but decrease in epileptic animals. Following KA or 3-NPA treatment, LAMA1 expression in vascular elements increases (Sarkar and Schmued, 2010). In pilocarpine-induced epilepsy, laminin expression rises on Days 3 and 4 post-SE but normalizes by Day 30 (Kim et al., 2014), consistent with our findings. Thus, expression patterns of laminin align with AQP4 distribution and pericyte vascular coverage during TLE progression and stabilizing extracellular matrix components like LAMA1 in pericytes could help delay epilepsy progression (Pitkänen et al., 2014). Matrix metalloproteinases (MMPs) are known to regulate neurovascular extracellular matrix stability and are involved in post-traumatic epileptogenesis, with MMP inhibitors reducing epileptogenesis (Ikonomidou, 2014; Jayakumar et al., 2017; Pijet et al., 2018). Notably, pericytes release the most MMP9 upon thrombin stimulation compared with endothelial cells and astrocytes (Machida et al., 2015), highlighting the potential role of pericyte-derived MMP9 in stabilizing extracellular matrix components like laminins and AQP4 polarity.

PDGF-BB/PDGFRβ signaling is crucial for vascularization by mediating pericyte recruitment to the vasculature, thereby promoting vessel integrity and function (Tsioumpekou et al., 2020). Previous studies have shown that exogenous PDGF-BB, administered 1–14 d post-KA injury, can maintain pericyte distribution, and inhibit epileptogenesis (Arango-Lievano et al., 2018). However, the dynamics of PDGF-BB during TLE remain unclear. In this study, we are the first to examine the changes in PDGF-BB from injury to seizure, finding that its levels gradually decrease in both blood and CSF as TLE progresses. These findings suggest that increasing PDGF-BB during the latency period after injury could potentially inhibit TLE epileptogenesis.

### Limitations of this study

One limitation of this study is the reliance on behavioral video monitoring to identify seizures. While this approach can reliably detect convulsive seizures, it may fail to capture nonconvulsive or subclinical events. Another limitation of this study is the use of trifluoperazine, which is a nonspecific calmodulin antagonist with known effects on multiple neurotransmitter systems. The observed changes in seizure parameters and AQP4 polarity may reflect indirect or off-target effects. Further studies using more selective modulators or genetic tools are needed to validate the role of AQP4 polarity in seizure activity. Third, the relatively small sample size (*n* = 3) in several experiments may limit the statistical power and affect statistical robustness.

### Conclusion

The vascular distribution of pericytes is closely associated with the polarization of AQP4 during TLE pathology. Stabilizing extracellular matrix proteins of pericytes, such as LAMA1 and LAMA2, and administering PDGF-BB post-injury to preserve pericyte distribution around blood vessels may help maintain AQP4 polarity and potentially contribute to slowing TLE progression.

## Data Availability

Sequence data that support the findings of this study have been deposited in the European Nucleotide Archive with the primary accession code PRJEB88623.

## Synthesis

Reviewing Editor: Mihaela Iordanova, Concordia University - Loyola Campus Decisions are customarily a result of the Reviewing Editor and the peer reviewers coming together and discussing their recommendations until a consensus is reached. When revisions are invited, a fact-based synthesis statement explaining their decision and outlining what is needed to prepare a revision will be listed below. The following reviewer(s) agreed to reveal their identity: Larissa Kraus.

Overall both reviewers found this paper interesting and addressing important questions in the molecular eitology of epilepsy. There are a number of significant concerns, however, that should be addressed.

Major issues:

- Were the same animals repeatedly given pentobarb to collect blood and tissue, or was that a terminal procedure? This would affect the type of statistical analysis used (single vs repeated measures) and could affect disease progression as pentobarb is anti-convulsant. This is not clear.

- Behavioral monitoring of seizures is not ideal and should be mentioned as a caveat of the study.

- What does "Day 0" represent in relationship to when pilocarpine is given? That's not clear.

- Why was pilocarpine in rats used for one part of the study but PTZ in mice was used in another?

- The conclusions drawn from the trifluoperazine data are overstated and should be removed from the paper or included with the many caveats they come with. Trifluoperazine is generally used as an antipsychotic and acts at dopamine and cholinergic systems. It has been reported to reduce Aqp4 mRNA and protein expression in a stroke model, but those mechanisms are likely not at play in the model used here because trifluoperazine was delivered simultaneously with PTZ. The authors should significantly reconsider these experiments and their many caveats. Furthermore, the statements "Thus, trifluoperazine suppressed PTZ-induced seizures, which was related to AQP4 polar distribution. AQP4 polarity is involved in seizures" should be removed. The data presented do not justify these conclusions.

-The method used to pick epileptic and non-epileptic rats is very confusing and not ideal. In some experiments animals that had SE but 30 days later had smooth coats were considered controls. This is not a rigorous way to identify epileptic vs non-epileptic animals. The author should strive to convey the system used to generate and select epileptic animals more precisely for every experiment.

- The labels in the bioinformatic analysis of RNAseq data are too small to read.

- Can the authors explain what they mean in the intro by "Pericytes did not affect dystrophin-associated protein components in astrocytes". This seems at odds with data presented in Fig. 4, 6, 7.

- Many studies are minimally powered, with n = 3 for multiple experiments. This caveat should be noted in the discussion.

- The overall conclusion that AQP4 polarity plays a crucial role in epilepsy development is not sufficient supported by the data to be included as a key conclusion. The data supporting this is correlational at best.

- TLE vs general epilepsy: Both pilocarpine and PTZ are established models for inducing temporal lobe epilepsy (TLE) and are therefore typically used to study TLE specifically, rather than epilepsy in general. The authors reference human TLE in the discussion (lines 341-342) to compare AQP4 polarization changes observed in their models with those reported in human cases. Additionally, their investigation focuses on the hippocampus, which is the primary seizure focus in TLE. However, the manuscript largely interprets and discusses the findings in the broader context of epilepsy, rather than specifically within the scope of TLE. This should be revised, as both the chosen animal models and the brain region studied are specific to TLE, and the conclusions should reflect this more accurately.

- Why were two different TLE models used in this study? It is unclear which animal models were used for specific experiments. Please provide precise descriptions in both the Methods and Results sections to clearly indicate which model was used for each experiment.

- Please include the full, uncropped images of the Western blots as a supplementary figure.

## Author Response

Synthesis of Reviews:

Synthesis Statement for Author (Required):

Overall both reviewers found this paper interesting and addressing important questions in the molecular eitology of epilepsy. There are a number of significant concerns, however, that should be addressed.

1. - Were the same animals repeatedly given pentobarb to collect blood and tissue, or was that a terminal procedure? This would affect the type of statistical analysis used (single vs repeated measures) and could affect disease progression as pentobarb is anti-convulsant. This is not clear.

Reply 1: Thank you for your valuable comment. We apologize for the lack of clarity. In our study, each animal was anesthetized at a single time point only-either at the end of SE, or at 1, 7, or 30 days post-modeling-for terminal blood and tissue collection. Thus, no animal underwent repeated anesthesia or sampling. We have revised the Methods section to clarify this. Please see Lines 104-106.

2. - Behavioral monitoring of seizures is not ideal and should be mentioned as a caveat of the study.

Reply 2: Thank you for your insightful comment. We agree that behavioral monitoring alone has limitations in accurately identifying all seizure events, especially subclinical or electrographic seizures. In our study, video-based behavioral observation was used to distinguish epileptic from non-epileptic animals based on observable convulsive events, which is a commonly used method in preclinical epilepsy models when EEG monitoring is not available. We have now acknowledged this limitation in the Discussion section, as follows: "One limitation of this study is the reliance on behavioral video monitoring to identify seizures. While this approach can reliably detect convulsive seizures, it may fail to capture non-convulsive or subclinical events. Future studies incorporating EEG monitoring would provide a more comprehensive assessment of seizure activity." Please see Lines 397-399.

3. - What does "Day 0" represent in relationship to when pilocarpine is given? That's not clear.

Reply 3: Thank you for pointing this out. In our study, "Day 0" refers to the time point immediately after the termination of SE, which was induced by pilocarpine administration. Blood and tissue samples were collected right after SE ended, which is typically several hours after pilocarpine injection. We have clarified this in the revised manuscript as follows: "0d represents the time point immediately after the termination of pilocarpine-induced SE." Please see Lines 208, 525-526.

4. - Why was pilocarpine in rats used for one part of the study but PTZ in mice was used in another? Reply 4: Thank you for your important question. We used pilocarpine-induced SE in rats and PTZ-induced seizures in mice to explore different aspects of seizure-related pathology. The pilocarpine model in rats is a well-established model of temporal lobe epilepsy with chronic spontaneous seizures, which was suitable for examining long-term neurodegenerative changes, including alterations in pericytes and astrocytes over an extended post-SE period. In contrast, the PTZ model in mice induces acute, chemically provoked seizures, and was employed to study the effects of modulating AQP4 polarity on acute seizure activity, due to its reproducibility and suitability for certain pharmacological manipulations (such as trifluoperazine here) in mice. PTZ-induced seizures were modeled in mice rather than rats due to their higher sensitivity to PTZ, better reproducibility of seizure induction. Please see Lines 78-79,113-114.

5. - The conclusions drawn from the trifluoperazine data are overstated and should be removed from the paper or included with the many caveats they come with. Trifluoperazine is generally used as an antipsychotic and acts at dopamine and cholinergic systems. It has been reported to reduce Aqp4 mRNA and protein expression in a stroke model, but those mechanisms are likely not at play in the model used here because trifluoperazine was delivered simultaneously with PTZ. The authors should significantly reconsider these experiments and their many caveats. Furthermore, the statements "Thus, trifluoperazine suppressed PTZ-induced seizures, which was related to AQP4 polar distribution. AQP4 polarity is involved in seizures" should be removed. The data presented do not justify these conclusions.

Reply 5: Thank you for this important and constructive comment. We fully agree that the conclusions drawn from the trifluoperazine experiments were overstated in the original manuscript. Trifluoperazine is a non-specific compound with known effects on dopaminergic and cholinergic signaling, and its acute administration may have multiple downstream effects beyond modulation of AQP4 polarity. We have now significantly revised the relevant paragraph to more cautiously describe our findings as associative observations rather than causal conclusions. In addition, we have removed the statements: "Thus, trifluoperazine suppressed PTZ-induced seizures, which was related to AQP4 polar distribution. AQP4 polarity is involved in seizures." The limitations of this experiment have also been added to the Discussion section. Please see Lines 224-227, 399-403.

6. -The method used to pick epileptic and non-epileptic rats is very confusing and not ideal. In some experiments animals that had SE but 30 days later had smooth coats were considered controls. This is not a rigorous way to identify epileptic vs non-epileptic animals. The author should strive to convey the system used to generate and select epileptic animals more precisely for every experiment.

Reply 6：Thank you for pointing this out. We agree that the original description lacked clarity and may have given the impression of subjective or inconsistent selection criteria. In our study, animals were continuously monitored via video for one week starting at 21 days post-SE to assess the presence of spontaneous recurrent seizures. Animals were classified as epileptic if they exhibited at least two unprovoked seizures with a minimum 24-hour interval-a standard definition for epilepsy.

For animals that did not display seizures during the video monitoring period, we acknowledge the limitation that late-onset、non-convulsive or subclinical seizures could have been missed. To further reduce this possibility in downstream experiments (e.g., PDGF-BB testing), we additionally selected animals that not only showed no seizures during monitoring but also exhibited a normal external appearance (normal grooming, smooth fur), similar to control animals. While this approach is empirical and indirect, it was used cautiously to increase the likelihood that selected animals truly lacked epileptic activity. We now clearly describe these criteria in the revised Methods section and acknowledge this limitation in the Discussion. Please see Lines 97-103, 244-246, 397-399.

7. - The labels in the bioinformatic analysis of RNAseq data are too small to read.

Reply 7: Thank you for your comment. We have enlarged the figure labels as much as possible within the layout constraints to enhance readability. We hope this revision addresses the issue. Please see Figure 5.

8. - Can the authors explain what they mean in the intro by "Pericytes did not affect dystrophin-associated protein components in astrocytes". This seems at odds with data presented in Fig. 4, 6, 7.

Reply 8: Thank you for pointing this out. We apologize for the confusion. The statement "Pericytes did not affect dystrophin-associated protein components in astrocytes" is derived specifically from our transcriptomic analysis (RNA-seq) of astrocytes co-cultured with or without pericytes. In this analysis, the mRNA levels of several key dystrophin-associated protein components in astrocytes - including α-syntrophin, agrin, dystrophin, β-dystroglycan, and α-dystrobrevin - were not significantly altered by pericyte co-culture.

In contrast, the results shown in Figure 4 indicate that pericyte co-culture increased membrane localization of AQP4 in astrocytes, without affecting total AQP4 expression. Since AQP4 itself is not considered a component of the dystrophin-associated protein complex, this result does not contradict our RNA-seq findings.

As for Figures 6 and 7, although LAMA1 and LAMA2 expression changed during epilepsy progression, we cannot determine whether LAMA1 or LAMA2 are primarily derived from pericytes or astrocytes based on existing experimental data.

Therefore, we have clarified in the manuscript that our statement regarding the lack of pericyte effects on dystrophin-associated proteins is strictly based on transcriptional data from astrocytes. We have now revised the sentence in the abstract to reflect this more accurately and avoid misunderstanding. Please see Lines 15-17, 276-280.

9. - Many studies are minimally powered, with n = 3 for multiple experiments. This caveat should be noted in the discussion.

Reply 9: Thank you for this important comment. We acknowledge that some of our experiments were conducted with a relatively small sample size (n = 3), which may limit the statistical power and generalizability of the findings. We have now added a statement in the Discussion section to address this limitation. Please see Lines 404-405.

10. - The overall conclusion that AQP4 polarity plays a crucial role in epilepsy development is not sufficient supported by the data to be included as a key conclusion. The data supporting this is correlational at best.

Reply 10: Thank you for your insightful comment. We fully acknowledge that our current data are primarily correlative and do not directly demonstrate a causal relationship. Accordingly, we have revised the abstract and conclusion to avoid overstating the causal role. Please see revised Lines 21-24, 408-412.

11. - TLE vs general epilepsy: Both pilocarpine and PTZ are established models for inducing temporal lobe epilepsy (TLE) and are therefore typically used to study TLE specifically, rather than epilepsy in general. The authors reference human TLE in the discussion (lines 341-342) to compare AQP4 polarization changes observed in their models with those reported in human cases. Additionally, their investigation focuses on the hippocampus, which is the primary seizure focus in TLE. However, the manuscript largely interprets and discusses the findings in the broader context of epilepsy, rather than specifically within the scope of TLE. This should be revised, as both the chosen animal models and the brain region studied are specific to TLE, and the conclusions should reflect this more accurately.

Reply 11: Thank you for your thoughtful and accurate observation. We acknowledge that both pilocarpine and PTZ are well-established models for TLE, and our investigation specifically focused on the hippocampus, a key region implicated in TLE. We have now revised the manuscript accordingly to reflect this specificity. Mentions of "epilepsy" in the title, abstract, introduction, models, results, discussion and conclusions have been revised to "TLE" to ensure consistency and accuracy. Where general epilepsy mechanisms are discussed in reference to the broader literature, we have clarified the context appropriately. Please see revisions in Lines 2, 6, 7, 41, 77, 113, 203, 229, 241, 307, et al..

12. - Why were two different TLE models used in this study? It is unclear which animal models were used for specific experiments. Please provide precise descriptions in both the Methods and Results sections to clearly indicate which model was used for each experiment.

Reply 12: Thank you for this valuable comment. We employed the pilocarpine-induced SE model in rats to investigate chronic TLE-related pathological changes, while the PTZ-induced seizure model in mice was used to study acute seizure dynamics and related interventions. We have now revised the Methods and Results sections to clearly specify which animal model was used for each experiment. Specifically, the pilocarpine model was employed for establishing chronic TLE and investigating the temporal changes in AQP4 polarity, pericyte vascular coverage and PDGF-BB while the PTZ model was used to study the effects of modulating AQP4 polarity on acute seizure activity and associated brain edema. Please refer to the revised manuscript Lines 78-79, 113-114, 204-205, 219-221, 230-231, 243-244, 293-295, 572 for detailed descriptions.

13. - Please include the full, uncropped images of the Western blots as a supplementary figure.

Reply 13: We thank the reviewer for this valuable suggestion. The full, uncropped Western blot images have been added as Extended Figures according to the journal's policy.
