# Supplementary figures and images for: The PDGFBB-PDGFRβ Pathway and Laminins in Pericytes Are Involved in the Temporal Change of AQP4 Polarity during Temporal Lobe Epilepsy Pathogenesis
Source: eNeuro. 2025 Oct 24;12(10):ENEURO.0196-25.2025. doi: 10.1523/ENEURO.0196-25.2025 (PMC12669438; doi:10.1523/ENEURO.0196-25.2025)

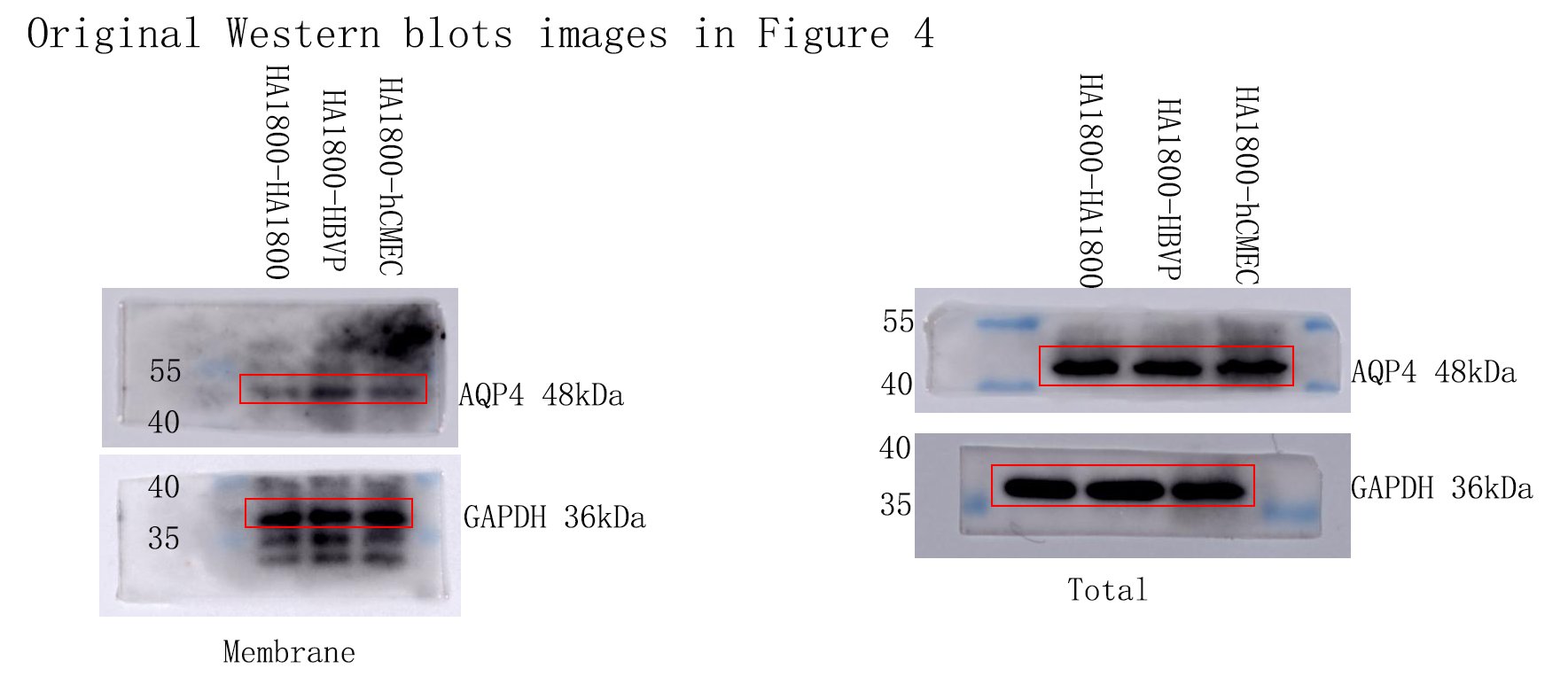

Supplement: Figure 4-1 — Original Western blots in Figure 4. The quantified bands correspond to the molecular weight indicated in the antibody datasheet of AQP4 and GAPDH. Download Figure 4-1, TIF file. [file eneuro-12-ENEURO.0196-25.2025-s002.tif]

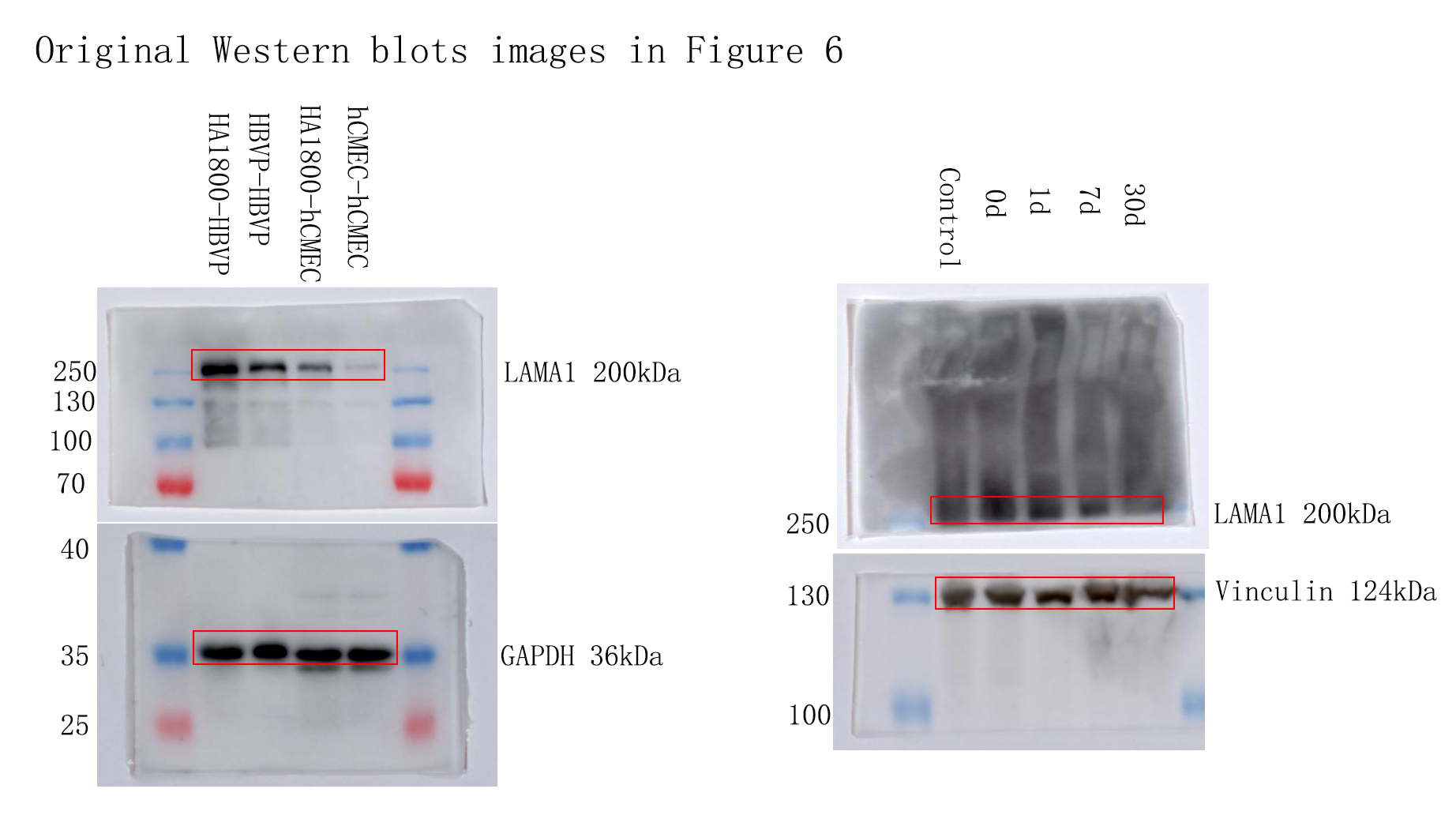

Supplement: Figure 6-1 — Original Western blots in Figure 6. The quantified bands correspond to the molecular weight indicated in the antibody datasheet of LAMA1. Download Figure 6-1, TIF file. [file eneuro-12-ENEURO.0196-25.2025-s004.tif]

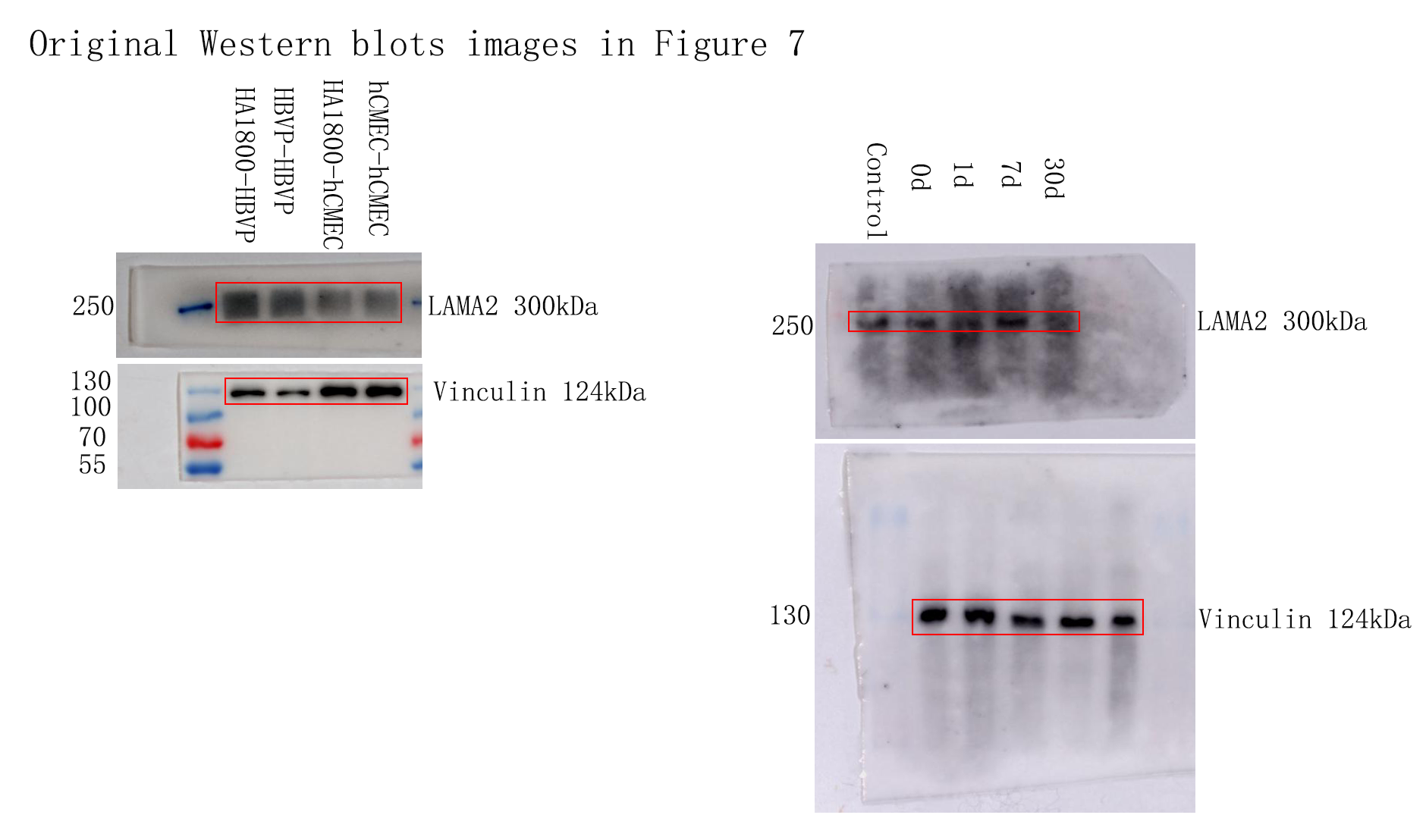

Supplement: Figure 7-1 — Original Western blots in Figure 7. The quantified bands correspond to the molecular weight indicated in the antibody datasheet of LAMA2. Download Figure 7-1, TIF file. [file eneuro-12-ENEURO.0196-25.2025-s005.tif]
